# GCGene: a gene resource for gastric cancer with literature evidence

**DOI:** 10.18632/oncotarget.9030

**Published:** 2016-04-26

**Authors:** Min Zhao, Luming Chen, Yining Liu, Hong Qu

**Affiliations:** ^1^ School of Engineering, Faculty of Science, Health, Education and Engineering, University of The Sunshine Coast, Maroochydore DC, Queensland, Australia; ^2^ Center for Bioinformatics, State Key Laboratory of Protein and Plant Gene Research, College of Life Sciences, Peking University, Beijing, P.R. China

**Keywords:** gastric cancer, database, cancer genomics, functional analysis

## Abstract

Gastric cancer (GC) is the fifth most common cancer and third leading cause of cancer-related deaths worldwide. Its lethality primarily stems from a lack of detection strategies for early stages of GC and a lack of noninvasive detection strategies for advanced stages. The development of early diagnostic biomarkers largely depends on understanding the biological pathways and regulatory mechanisms associated with putative GC genes. Unfortunately, the GC-implicated genes that have been identified thus far are scattered among thousands of published studies, and no systematic summary is available, which hinders the development of a large-scale genetic screen. To provide a publically accessible resource tool to meet this need, we constructed a literature-based database GCGene (Gastric Cancer Gene database) with comprehensive annotations supported by a user-friendly website. In the current release, we have collected 1,815 unique human genes including 1,678 protein-coding and 137 non-coding genes curated from extensive examination of 3,142 PubMed abstracts. The resulting database has a convenient web-based interface to facilitate both textual and sequence-based searches. All curated genes in GCGene are downloadable for advanced bioinformatics data mining. Gene prioritization was performed to rank the relative relevance of these genes in GC development. The 100 top-ranked genes are highly mutated according to the cohort of published studies we reviewed. By conducting a network analysis of these top-ranked GC-associated genes in the human interactome, we were able to identify strong links between 8 highly connected genes with low expression and patient survival time. GCGene is freely available to academic users at http://gcgene.bioinfo-minzhao.org/.

## INTRODUCTION

Gastric (stomach) cancer (GC) is the fifth most commonly diagnosed cancer (952,000 new cases diagnosed in 2012) and the third leading cause of cancer-related deaths in both sexes worldwide [1]. In the United States, approximately 10,720 people died of GC (6,500 men and 4,220 women) in 2015 [2]. The majority of GC cases (70%) occur in developing countries, and half all cases occur in eastern Asia, mainly in Korea, Mongolia, Japan, and China [1].

Despite ongoing efforts to develop effective treatments, the 5-year survival rate of GC patients is only 29% [2]. As a heterogeneous disease, GC has complex molecular mechanisms for uncontrolled cell growth, which could be caused by promoter methylation [3], deregulated gene expression [4], competing endogenous long non-coding RNAs [5, 6], and/or copy number alteration of tumor-suppressor genes and oncogenes [7]. The majority of GC studies to date have not focused beyond the gene level; thus, they fail to provide the whole picture of tumorigenesis. In this study, we aimed to develop the first literature-based genetic resource with extensive annotations, GCGene. This data resource can also be used to prioritize genes by their GC-associated importance relevance and to identify both the common and unique cellular events at different oncogenic stages.

## RESULTS AND DISCUSSION

To survey the genetic information related to all GC types, we performed extensive data integration and literature curation. Ultimately, we identified 1,815 non-redundant GC-associated genes for inclusion in this database, and we conducted functional annotation and gene prioritization of these genes (Table S1).

### Database construction and content

#### Web interface

**Figure 1:**
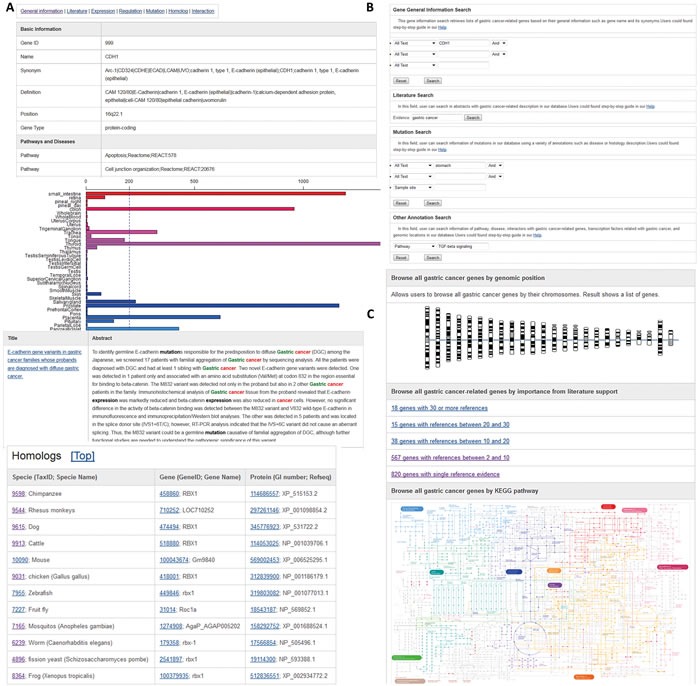
Web interface of GCGene **A.** The basic information in each GC-associated gene page; **B.** Query interface for text search; **C.** Browsing the genes in GCGene using chromosomes, literature supports, and KEGG pathways.

Based on the systematic survey of GC-associated genes in publically available databases and literature, we developed a user-friendly web interface to make this annotated information freely available to all researchers. The database is supported by a web browser that allows researchers to explore all the GC-associated genes using chromosome and coloured KEGG pathway maps (Figure 1). GCGene allows users to conduct quick queries by GeneID or gene symbol and to run BLAST searches against all human sequences. For advanced integrative study, a list of all genes curated in this database is available for download.

#### Information on the GCGene gene page

To organize information for each gene, we classified our annotation details into seven groups: general information, literature, gene expression, regulation, genetic mutation, homologous gene, and protein-protein interaction. When exploring the annotations, users can click the labels at the top of each web page to reach any specific annotation quickly (Figure 1). On the general information page for each gene, the official gene symbol, alias, biological function, involved biological phenotype, and nucleotide/protein sequences are listed. The cross-references to other public bioinformatics databases such as TSGene [8, 9], NCBI Entrez Gene [10], dbEMT [11], iHOP [12], and MSGene [13] are included. All available literature associated with a gene is highlighted with cancer keywords. A comprehensive gene-expression profile of normal and tumor samples plotted from BioGPS is provided [14]. On the regulation page, the transcription factor, post-translational modification, and methylation are annotated. Homologous sequences from other model species have also been integrated from the NCBI HomoloGene database [15]. On the mutation page, the genetic polymorphisms from the most updated COSMIC database (version 73) have been collected [16]. Finally, the protein-protein interaction data from the PathwayCommons database (version 6) have been grouped into physical interactions, signaling interactions, and metabolic interactions [17].

#### Browsing the classified genes in GCGene

GCGene supports a variety of ways to browse putative GC genes, including highlighted KEGG maps and chromosome distribution maps. The genomic distribution of all the genes has been plotted on 24 chromosomes with individual charts (Figure 1). Users can browse each chromosome to access all of the GC-associated genes in the region. The number of literature citations for each gene is provided, indicating its relative importance in GC development. To provide access to this information, we have included a browsing function that identifies different gene sets based on the number of literature citations.

#### Keyword-based search in GCGene

A search function at the top right corner of each web page can be used to conduct quick queries using human gene official symbols or Entrez Gene IDs. Advanced searches can be conducted by typing the gene name or its functional characteristics, including chromosome location, interaction partner, biological process, or disease (Figure 1). In addition, users can search all the curated literature by key words, which is useful in identifying candidate genes for specific biological processes.

#### Sequence-based search in GCGene

With the BLAST interface, users can evaluate gene sequence similarity by inputting the sequence of interest. The sequence alignment option can be modified with an E-value and an identity score. This database also facilitates bulk downloads of all nucleotide and protein sequences in a FASTA format for advanced local-sequence-based BLAST search (Figure 1).

### Gene ranking for all the genes in GCGene

Small-scale studies of GC often focus on verifying specific functions of cancer genes under a certain genetic background or other phenotype characteristic. Because hundreds of genes are associated with GC, it is necessary to systematically prioritize the most informative genes and to systematically construct a large-scale gene map for GC. Using the ToppGene gene-ranking tool [18], we prioritized the relative importance of all 1,815 genes in GCGene. To build a ranking model using ToppGene, we defined a training set with the 18 most commonly studied genes with ≥30 literature citations: *CDH1, CXCL8, ERBB2, GSTM1, GSTP1, GSTT1, HIF1A, IL10, IL1B, IL1RN, MTHFR, PTGS2, RUNX3, TNF, TP53, TYMS, VEGFA,* and *XRCC1*. ToppGene utilizes integrated biological annotation data to extract biological features from the training set to rank the remaining genes. Those biological annotations include protein domain, gene ontology evidence, pathway annotations, gene coexpression, sequence features, and other data mined from the literature. Finally, ToppGene was used to combine all the rankings into a global ranking for all the candidate GC genes using order statistics (Table S2). Not surprisingly, those 100 top-ranked genes are enriched in the cancer pathways identified with terms such as “regulation of cell proliferation,” “pathways in cancer,” “PI3K-Akt signaling pathway,” and “proteoglycans in cancer” (Table S3).

### Mutational patterns of the most relevant genes in GCGene

**Figure 2:**
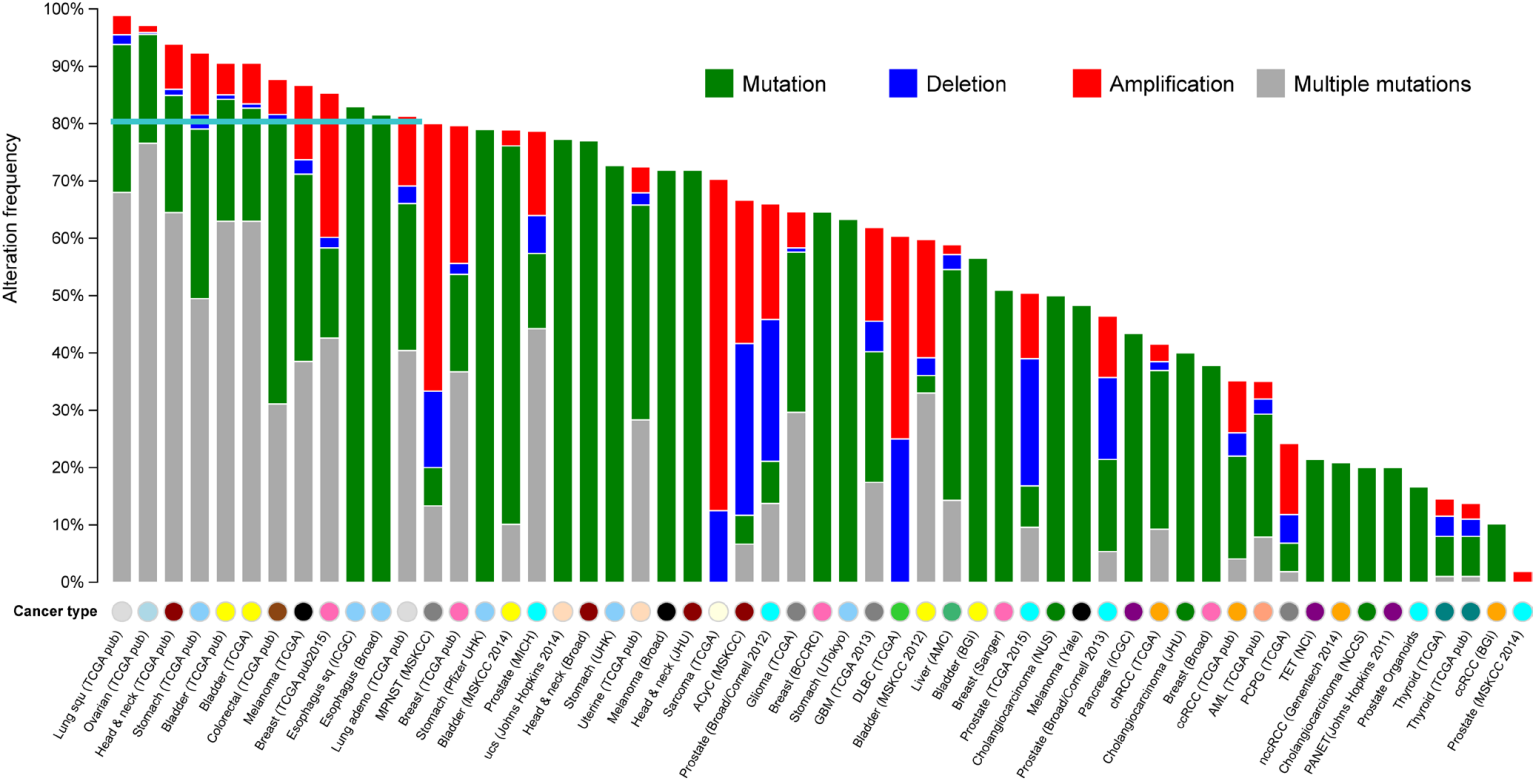
The mutational landscape for the 100 top-ranked GC-associated genes in multiple cancers The X axis represents the cancer types in TCGA, the Y axis represents the alteration frequencies of top 100 genes that correspond to the cancers on the X axis. The different colors indicate different cancer types based on their original tissues.

We systematically examined the 100 top-ranked genes in GCGene according to their somatic mutational patterns in multiple cancers using cBio portal [19]. These patterns are useful for the identification of highly mutated genes in other cancer types for further screening. As shown in Figure 2, a wide variety of genetic alterations (i.e., mutations, deletions, amplifications, and multiple alterations) in the 100 top-ranked genes occur in ≥80% of cases among 12 cancer cohorts. Notably, the majority of genes associated with these 12 cancers have multiple genetic alterations; therefore, some genes have both a single-nucleotide variation and a copy-number variation.

It is not surprising that multiple genetic mutations occur most frequently in cancers with higher alteration frequencies. With unique single-nucleotide mutations, deletion and amplification are more common in cancers with lower alteration frequencies. In our analysis, lung squamous-cell carcinoma had the highest overall gene alteration rate. Of the 176 cases studied, 98.9% showed genetic mutations of the genes; nearly 70% presented multiple alterations. TCGA ovarian serous cystadenocarcinoma and head and neck cancer had very similar rates of alteration. TCGA gastric adenocarcinoma had the fourth highest rate of mutation of these genes, 92.3%. The other two gastric adenocarcinomas have similar mutation frequencies in terms of single-nucleotide mutations. According to the datasets from Pfizer and UHK, stomach adenocarcinoma had 79% and 72.7% mutational frequency in 79 cases and 16 cases, respectively. This relatively lower rate of penetration might be explained by the small sample size. However, these three GC datasets show similar rates of single-nucleotide mutation.

### Networking the top-ranked genes to identify the hub genes associated with patient survival time

Recent advances in high-throughput technologies have dramatically increased the availability of protein-protein interaction (PPI) data and have stimulated the modelling of pathways to improve our understanding specific cellular events at the systems level. To avoid the high level of noise, sparseness, and highly skewed degree distribution of PPI networks, we utilized only reliable human PPIs summarized in a few popular biological pathway resources such as the KEGG and Reactome databases [20].

**Figure 3:**
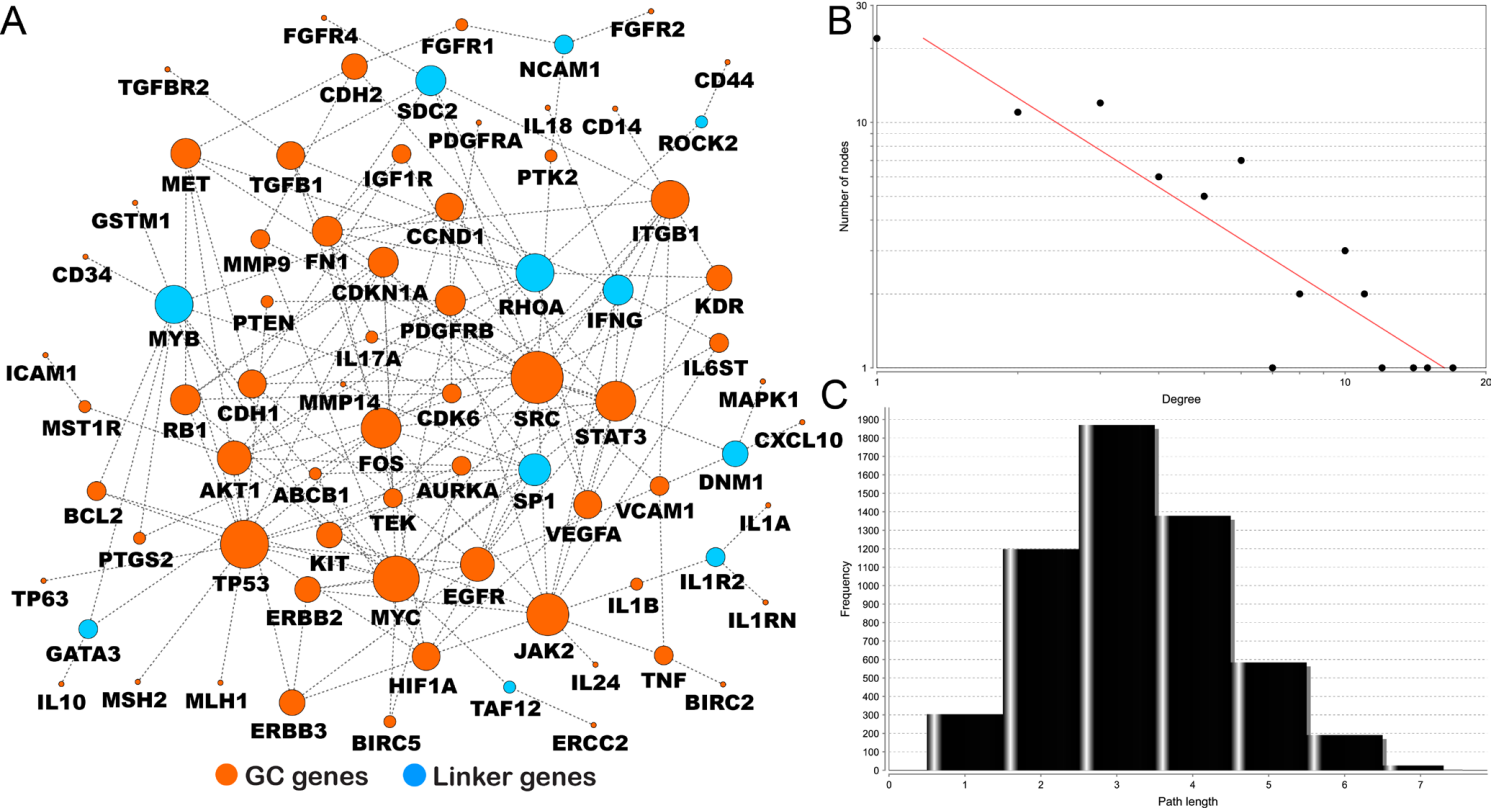
GC interactome using pathway-based protein-protein interaction data **A.** The 65 genes in orange are the 100 top-ranked genes in our GCGene; the remaining 11 genes in blue are linker genes that connect the 65 genes. The size of the node represents the number of connections in the network; **B.** the degree distribution; and **C.** the short path length frequency.

Using a search module [21], we extracted a subnetwork from all the human pathway-based interactomes. The reconstructed GC interactome contains 76 genes and 152 gene-gene interactions based on current evidence from known biological pathways (Figure 3A). Of the 76 nodes, 65 are among the 100 top-ranked GC-associated genes. The remaining 11 are genes that may potentially bridge the top-ranked GC-associated gene to fully implement its cellular function. The majority of GC genes are linked to each other in a highly modular structure. This finding not only supports the accuracy of our data but also shows that the GC genes are highly interconnected and form a high-density cellular modulus.

Further network topological analysis also revealed that most molecules in our map are closely connected. The degrees of connection of all nodes in our reconstructed GC map follow a power law distribution *P*(*k*)*~k^−b^*, where *P*(*k*) is the probability that a molecule has connections with other *k* molecules and *b* is an exponent with an estimated value of 1.207 (Figure 3B). Thus, our GC map is different from all the human PPI networks in which most nodes are sparsely connected, with an average *b* value of 2.9 [22]. We developed this feature to map the distribution of the shortest pathways throughout the entire network. This map revealed smaller pathway degrees (2 to 4), which means that majority of the node connections can be reached in only three steps on average (Figure 3C).

With dense interactions, the highly connected nodes in this network may have prominent roles as common connections that mediate information transduction in the short pathways. In total, we identified 9 genes with at least 10 connections: *SRC* (17), *TP53* (15), *MYC* (14), *JAK2* (12), *STAT3* (11), *FOS* (11), *RHOA* (10), *MYB* (10)*,* and *ITGB1* (10). Interestingly, *SRC* is the most connected node. Notably, only *MYB* has not been reported to be involved in GC in these 9 genes.

**Figure 4:**
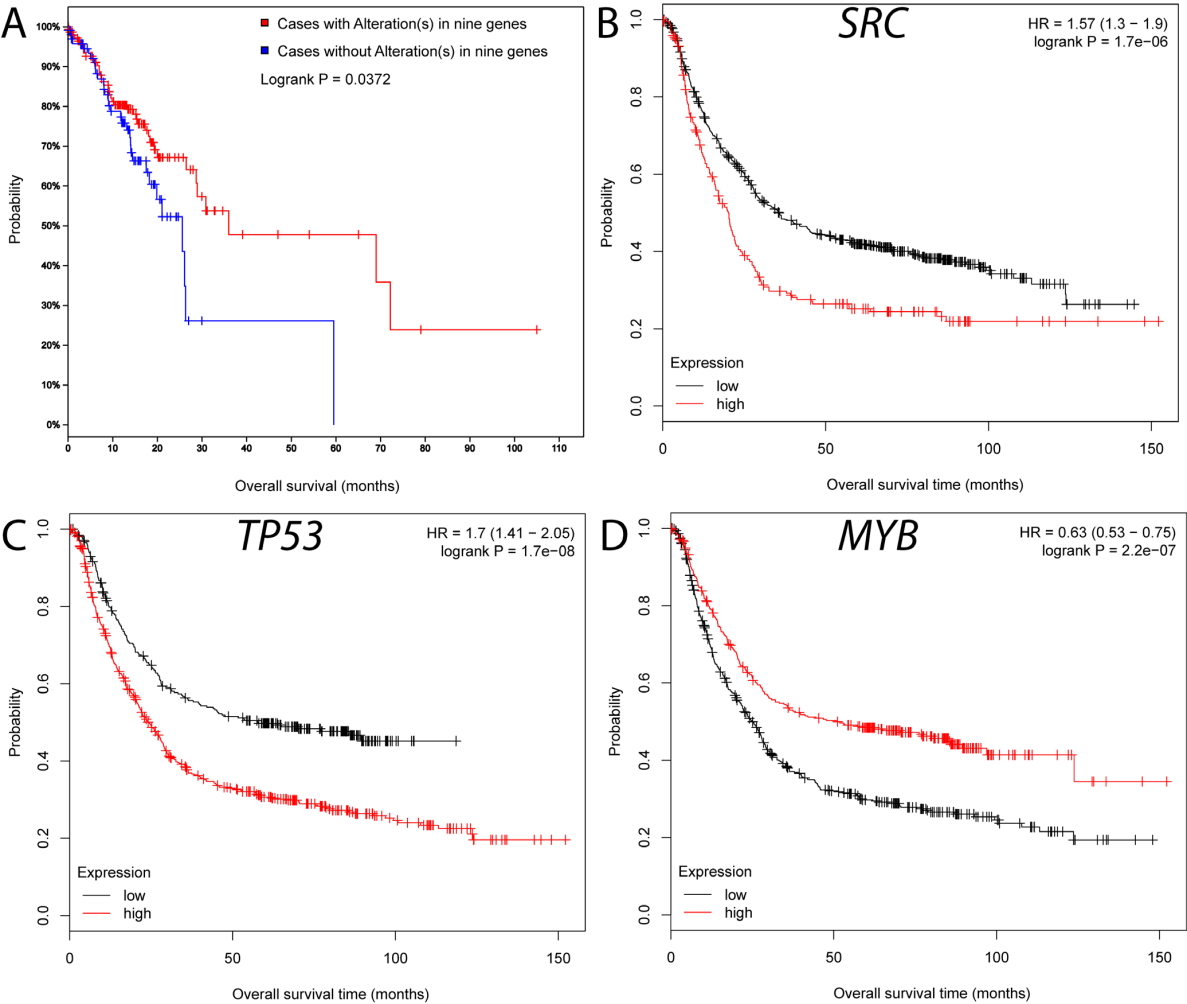
Survival analyses for the hub genes in the GC interactome **A.** Survival characteristics of the nine hub genes on the genetic mutation using cBio data portal [19]. **B.**-**D.** Survival characteristics of *SRC* (B), *TP53* (C) and *MYB* (D) based on the integrated GC gene expression using a KM plot [23].

We performed a survival analysis based on published TCGA mutational data using the cBio portal. Patients with genetic mutations in any of the 9 genes are significantly correlated with overall survival (Figure 4A). Further survival analyses using gene expression data also confirmed the importance of the 9 genes [23]. We found that lower expression of 8 genes (upper quartile vs. remaining samples) except *STAT3* is significantly correlated with longer relapse-free survival (*P* ≤ 0.05) (Figure 4B-4D, Figures S1-S6). In particular, the *MYB* is mutated in 2% of the TCGA GC cohort, and it is also associated with survival (Figure 4D). Taken together, these results highlight the potential role of *MYB* in GC progression. In summary, our reconstructed map not only reveals multiple hubs related to survival but also provides a broader context for the previously unconnected GC genes.

## CONCLUSIONS

In conclusion, our systematic curation of genetic information related to GC yielded 1,815 putative human genes (1,678 protein-coding and 137 non-coding genes) for inclusion in the GCGene database. A user-friendly web interface was developed to provide access to all the genes, with additional gene annotation and literature information. As the first literature-based gene database for GC, GCGene provides a novel resource for researchers performing high-throughput genetic and clinical tests to identify GC-associated genetic variants.

To facilitate subsequent literature updates, we constructed an automatic literature search scheme using the “My NCBI” tool, which will return the relevant literature every two weeks. We will use the document similarity clustering method in Entrez to group the newly available articles to assist with literature curation. Additionally, to keep pace with the rapid growth of cancer genome data, we have built an automated system capable of importing functional information from various public data sources, which will enable us to integrate more annotations quickly. Once the data is updated, the web page will be updated accordingly on an annual basis.

## MATERIALS AND METHODS

### Data integration from existing bioinformatics recourses

**Figure 5:**
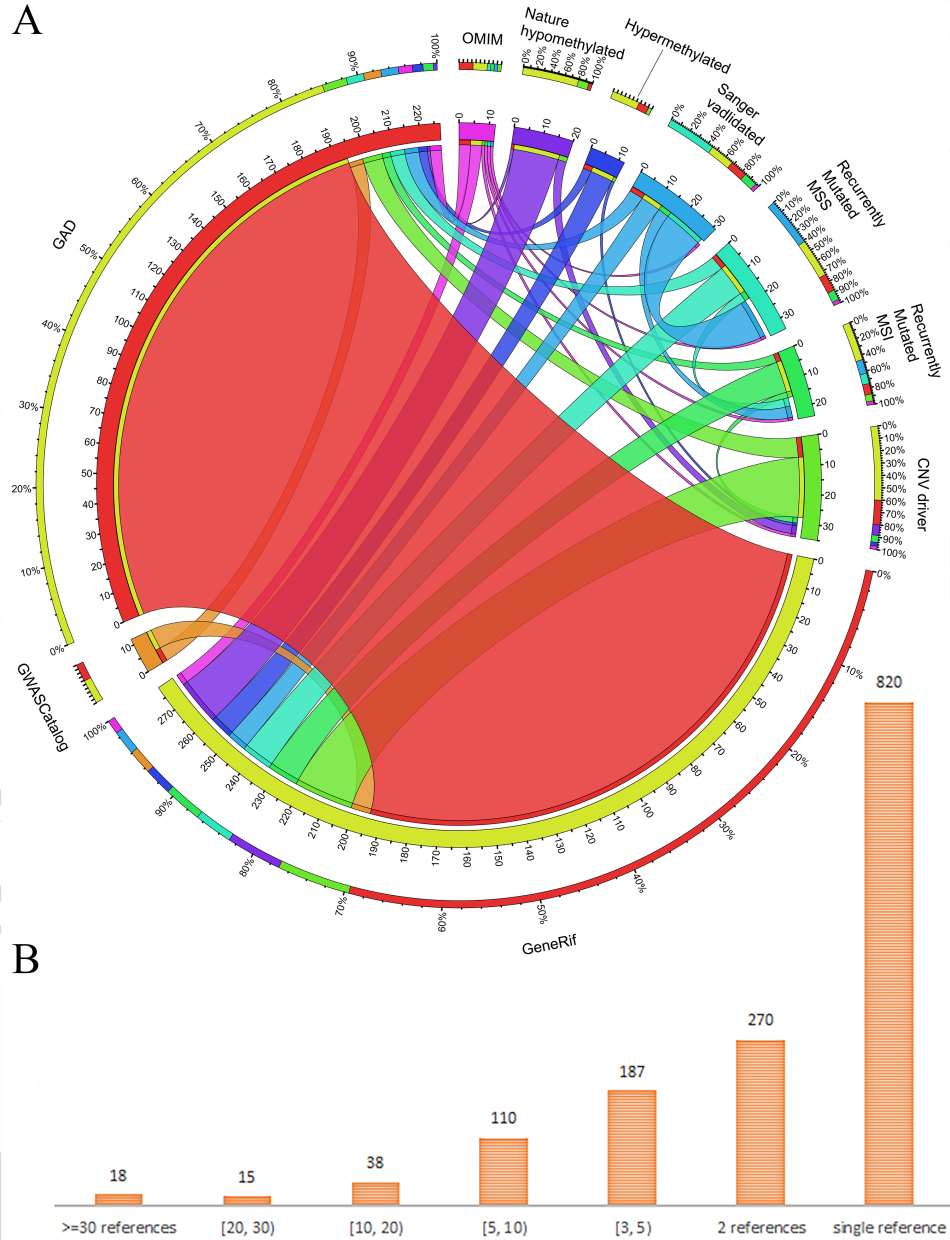
Overlapping of genes from different data sources and statistics **A.** Shared GC-associated genes across multiple data sources. The length of circularly arranged segments is proportional to the total genes in each regenerative process group. The ribbons connecting different segments represent the number of shared genes between regenerative process groups. The outer ring has stacked bar plots that represent the relative contribution of other regenerative process group to the regenerative process group totals. Ribbons connecting different segments represent the number of shared genes between regenerative tissues; **B.** statistics for number of genes with various pieces of literature evidence.

The gene collection related to GC was mainly based on 10 data sources: the OMIM (Online Mendelian Inheritance in Man, download on January 25, 2015) [24], GAD (The Genetic Association database, latest version updated on August 18, 2014) [25], gene manually curation from GeneRIF (Download on January 25, 2015) [26], genome-wide association studies from GWASCatalog (Download on January 25, 2015) [27], and 6 candidate gene lists produced by a large-scale genome-wide methylation and genetic mutation study on GC [28] (Figure 5A). As the most authoritative compendium of human disease-associated genes, OMIM does not include many genes. We obtained only 4 genes (*IL1B*, *IL1RN*, *KRAS*, and *CDH1*) associated with hereditary diffuse GC from OMIM. The GAD database is an archive of published human genetic association studies that contains curated information on candidate genes. In total, we collected 279 unique human genes from GAD from 637 published studies. In addition, 11 candidate genes were downloaded from 3 genome-wide association studies in the GWASCatalog database. In 2014, a whole-genome sequencing and comprehensive molecular profiling of GC identified numerous new driver mutations, including recently mutated genes of the microsatellite instability type (91 genes) and microsatellite-stable type (53 genes), Sanger sequencing was used to validate driver mutations (18 genes), genes within driver copy number variation regions (102 genes), as well as genes in hypermethylated (91 genes) and hypomethylated (92 genes) regions. We combined these publically available resources and harvested a list of 590 nonredundant human genes.

### Literature collection and gene curation

To assemble a detailed and precise GC gene resource with literature evidence, we performed an extensive literature query of GeneRIF database on January 10, 2015, using Perl regular expression to identify sentences with both gastric and cancer keywords: [(gastric OR stomach) AND (cancer OR tumor OR carcinoma)]. In total, we retrieved 2,904 PubMed abstracts. GeneRIF (Gene Reference Into Function) is a collection of short descriptions of gene functions in the Entrez Gene database [29]. However, GeneRIF records do not provide full abstracts for further curation. Thus, we downloaded all 2,904 PubMed abstracts in Medline format for manual review.

The curation of GC genes from literature in this study was conducted in three major steps: (1) grouping all 2,904 retrieved abstracts based on their semantic similarity using the “Related Articles” function in Entrez; (2) extracting contents related to GC from grouped abstracts; and (3) manually collecting gene names from the descriptions of the text and mapping the gene names to Entrez gene IDs. These curation steps allowed us to quickly and easily cross-check whether and how the curated abstract was related to GC. To provide a unified functional annotation, we used Entrez gene IDs as the key in all the tables of our GCGene database to cross-link the same genes from different public bioinformatics databases. To ensure the accuracy of our literature evidence, we collected the species information and the gene alias and manually mapped them to the official HUGO gene symbol. For example, in the sentence “Results suggest that the COX-2/microsomal prostaglandin E synthase-1 pathway contributes to the Helicobacter-associated gastric tumorigenesis,” [30] the gene COX-2 was one of the synonyms for the murine gene *Ptgs2* in the Entrez gene database. After careful manual cross-checking, we mapped all the curated genes to their corresponding human homologous groups using the NCBI HomoloGene database using the same method we implemented in a previous analysis [9, 31, 32]. In total, we identified 1,369 human homologous genes using Entrez. By integrating 590 genes from other public databases, we consolidated 1,815 human genes, 1,678 protein-coding and 137 non-coding genes (Table S1). The overlapping relationship among different data sources revealed that ~70% of genes from our literature content curation are also recorded in the GAD database (Figure 5A). These comparisons validated the high quality of our literature curation as well as multiple items of supporting evidence. Based on the curated references, we identified 18 genes with ≥30 supporting references (Figure 5B). The majority of the genes from literature curation (820 of 1,369 GC-associated genes; 59.90%) had only a single literature reference (Figure 5B), which may indicate the need for further experimental validation for these 820 candidate genes.

### Biological functional annotations

Information regarding comprehensive biological functional was retrieved from public resources for the annotation of the 1,815 human GC-associated genes in our database. The basic gene information and sequences were collected for each gene from the following databases: NCBI Entrez gene (downloaded on February 28, 2015) [33], UniProt (released February 2015) [34], Ensembl (version 78) [35], and Gene Ontology (downloaded on February 28, 2015) [36]. From BioGPS (downloaded on February 28, 2015) [14], the mRNA expression profiling data from both normal and tumor tissues were acquired from BioGPS (downloaded on February 28, 2015) [14]. The comprehensive pathway-associated information was annotated for GC-associated genes from the following databases: the transporter substrate database (version 1.0) [37], BioCyc (downloaded on February 28, 2015) [38], KEGG Pathway (downloaded on February 28, 2015) [39], the rate-limiting enzyme database (version 1.0) [40], PANTHER (downloaded on February 28, 2015) [41], PID Curated (downloaded on February 28, 2015) [42], the pathway localization database (version 1.0) [43], and Reactome (downloaded on February 28, 2015) [44, 45]. Disease information was imported from GAD (gene association database), KEGG Disease, Fundo (downloaded on February 28, 2015) [46, 47], NHGIR (downloaded on February 28, 2015) [48], and OMIM [33].

The original published GC-associated articles in PubMed were hyperlinked to their respective genes. Using the Perl Script and Swiss knife modules, functional information was integrated from Gene annotations [49], Gene Ontology annotations [36], HPRD/BIND/BioGRID interaction annotations, KEGG LIGAND/BioCarta (downloaded on February 28, 2015) signaling event annotations [50, 51], and OMIM annotations.

### Gene set enrichment analysis

The functional enrichment analysis of disease, pathways, and other functional annotations for each gene was accomplished using ToppFun [18]. In these analyses, the encoding genes of all human proteins were used as background, and the statistical significance of enriched annotations was calculated using the hypergeometric model. Based on the Benjamini-Hochberg multiple correction method in ToppFun, the corrected P-values for enriched annotations were calculated. Finally, the enriched annotations with corrected *P*-values < 0.01 were identified as over-representative annotations for each gene set. The resulting enriched gene ontology terms were further summarized and visualized by the REVIGO online server [52].

### Gene ranking using ToppGene and cancer mutation landscape

Using the ToppGene gene ranking tool [18], we prioritized the relative importance of each of the 1,815 GC-associated genes. ToppGene integrates the following biological annotation data to rank the input genes: protein domain, gene ontology evidence, pathway annotations, gene co-expression, sequence features, and data mined from the literature. First, ToppGene requires a training set, which includes most commonly studied genes associated with the biological processes of interest. In the present study, the training set consisted of 18 genes, each with ≥30 literature citations. This training set was used to extract features shared by all GC-associated genes.

Next, based on the extracted biological features from the training set, ToppGene builds a ranking model. The ranking model that contains multiple dimensional data is then used to prioritize the remaining 1,797 genes. Finally, the ToppGene ranking model combines all of the rankings into a global ranking for the 1,815 GC-associated genes using order statistics (Table S2). In the present study, the 100 top-ranked GC-associated genes were then input into the cBio portal to obtain a mutation pattern across multiple cancers.

### Construction of protein-protein interactome for the 100 top-ranked GC-associated genes

To study the potential biological mechanisms related to GC-associated genes, we extracted protein-protein interactions between the 100 top-ranked GC-associated genes and other human genes. To accomplish this task, we first collected a list of non-redundant pathway-based human interactomes from the PathwayCommons database, which includes several biological pathway databases such as KEGG and Reactome. We then extracted a subnetwork containing the 100 top-ranked GC-associated genes from the human interactome using an approach similar to the one implemented in our previous study [21]. All of the input genes were mapped into the human interactome using the proposed algorithm, and the subnetwork was extracted according to the shortest pathways between the input genes and other genes.

If the function of genes is systematically studied from the point of view of the network, the complexity and interconnectedness of the biological network is revealed. In general, biological networks tend to follow some simple rules, and the topological properties of the networks may be closely related to their function [53]. Therefore, we used the NetworkAnalyzer plug-in in Cytoscape 2.8 [54] (Figure 3B and 3C) to analyze the extracted subnetworks of GC-associated genes. We used degree to represent the sum of the number of connections for each node in a network [53], and the shortest path represented by the least number of steps from one node to another [53]. Cytoscape 2.8 was used to visualize the network.

## SUPPLEMENTARY MATERIALS FIGURES AND TABLES









## Abbreviations

TCGA: The Cancer Genome Atlas
GEO: Gene Expression Omnibus
GCGene: Gastric Cancer Gene database.
