# Supplementary material for: GCGene: a gene resource for gastric cancer with literature evidence
Source: Oncotarget. 2016 Apr 26;7(23):33983–93. doi: 10.18632/oncotarget.9030 (PMC5085132; doi:10.18632/oncotarget.9030)
Supplement: Supplementary file 1 [file oncotarget-07-33983-s001.pdf]

GCGene: a gene resource for gastric cancer with literature evidence

Supplementary Material

Table S1: The basic annotations of all curated 1815 GC-associated genes.

Table S2: The gene ranking results of GC-associated genes.

Table S3: The enriched biological pathways of the 100 top-ranked GC-associated genes.

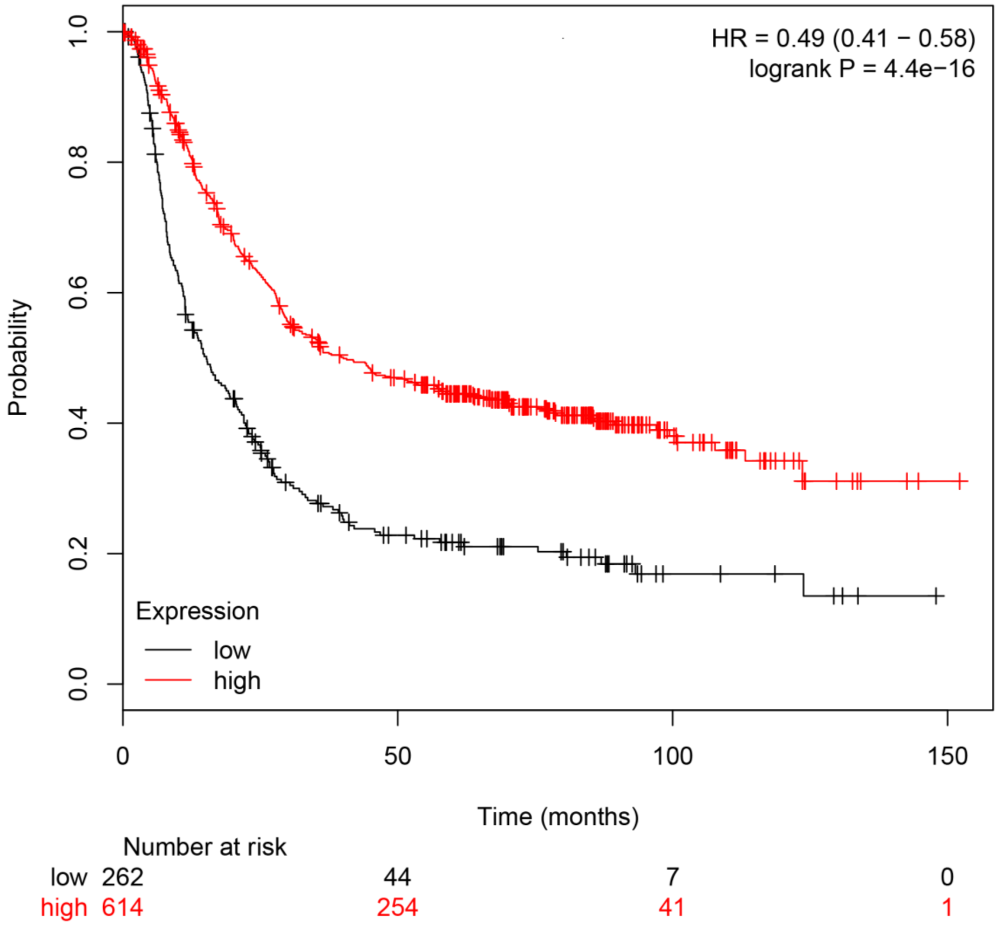

Figure S1: The survival analysis on *FOS*.

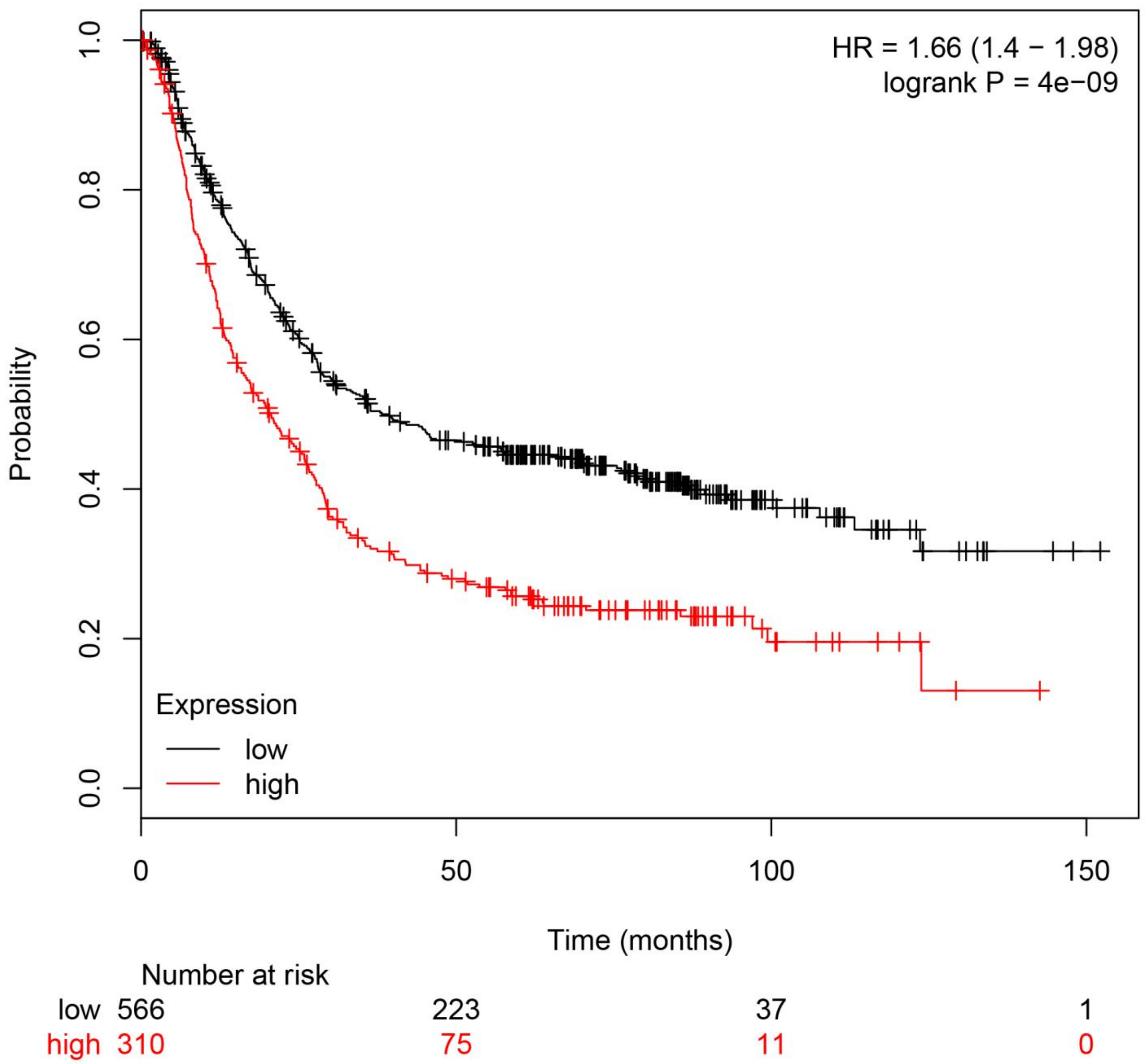

Figure S2: The survival analysis on *ITGB1*.

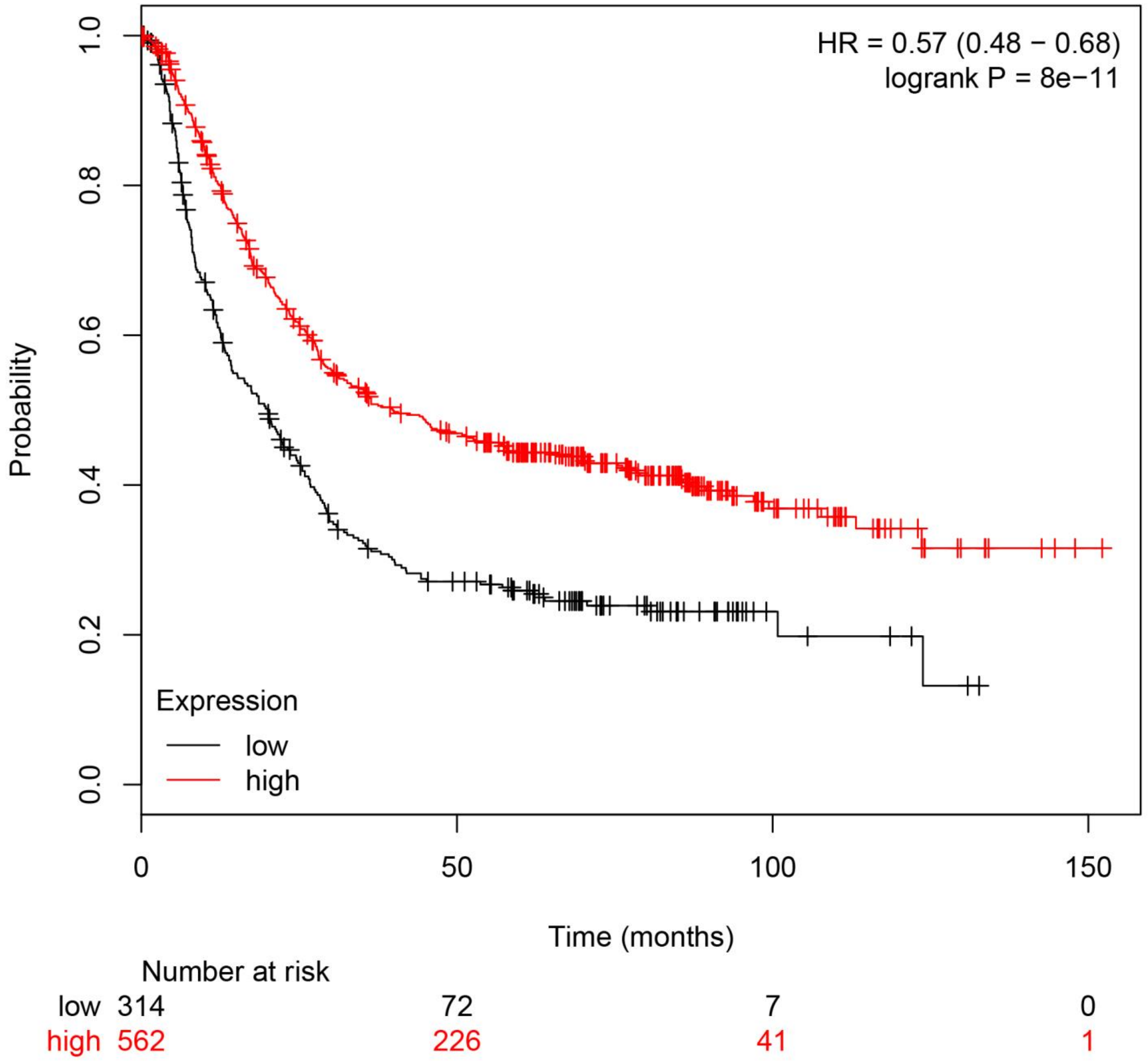

Figure S3: The survival analysis on *JAK2*.

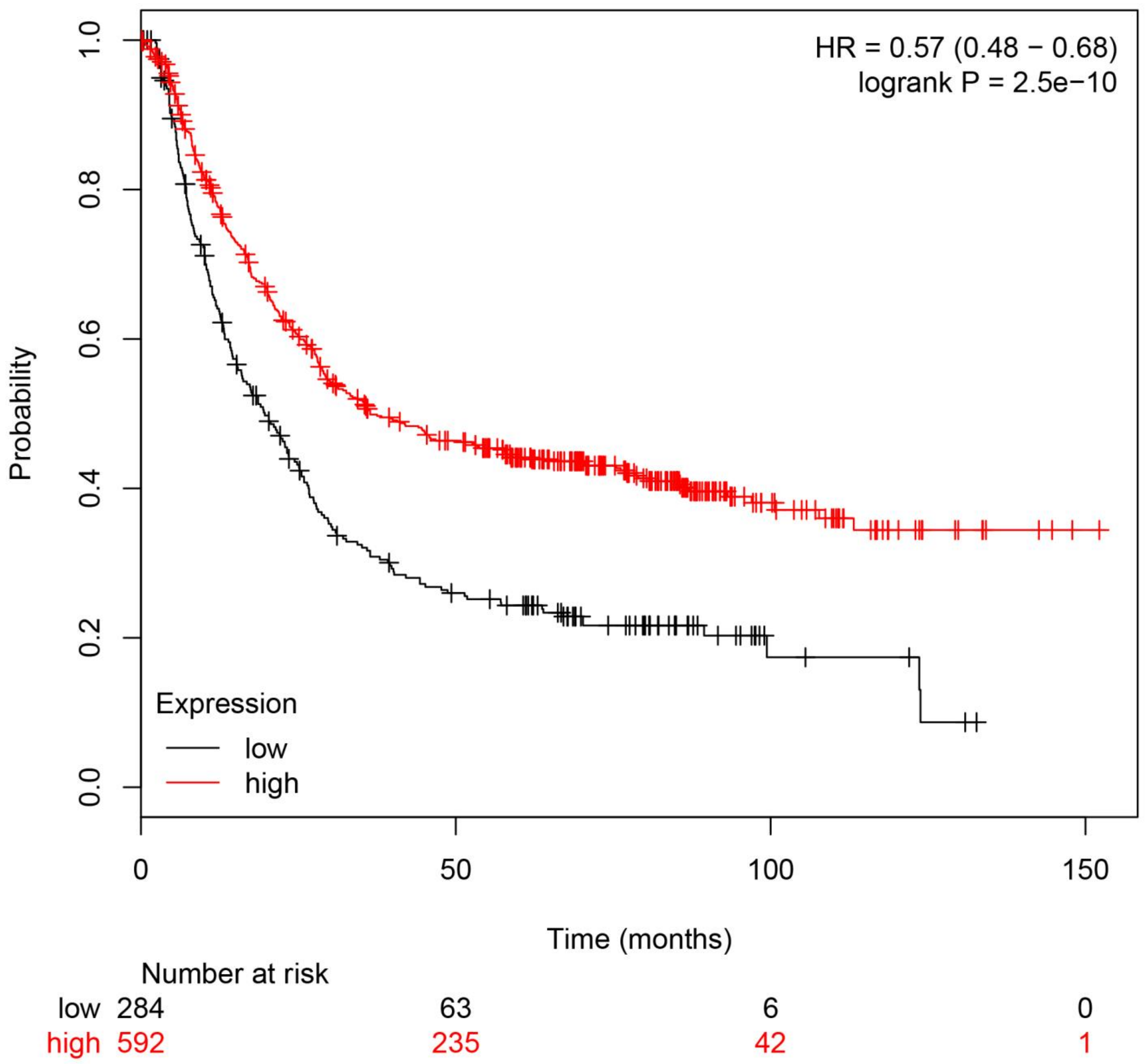

Figure S4: The survival analysis on *RHOA*.

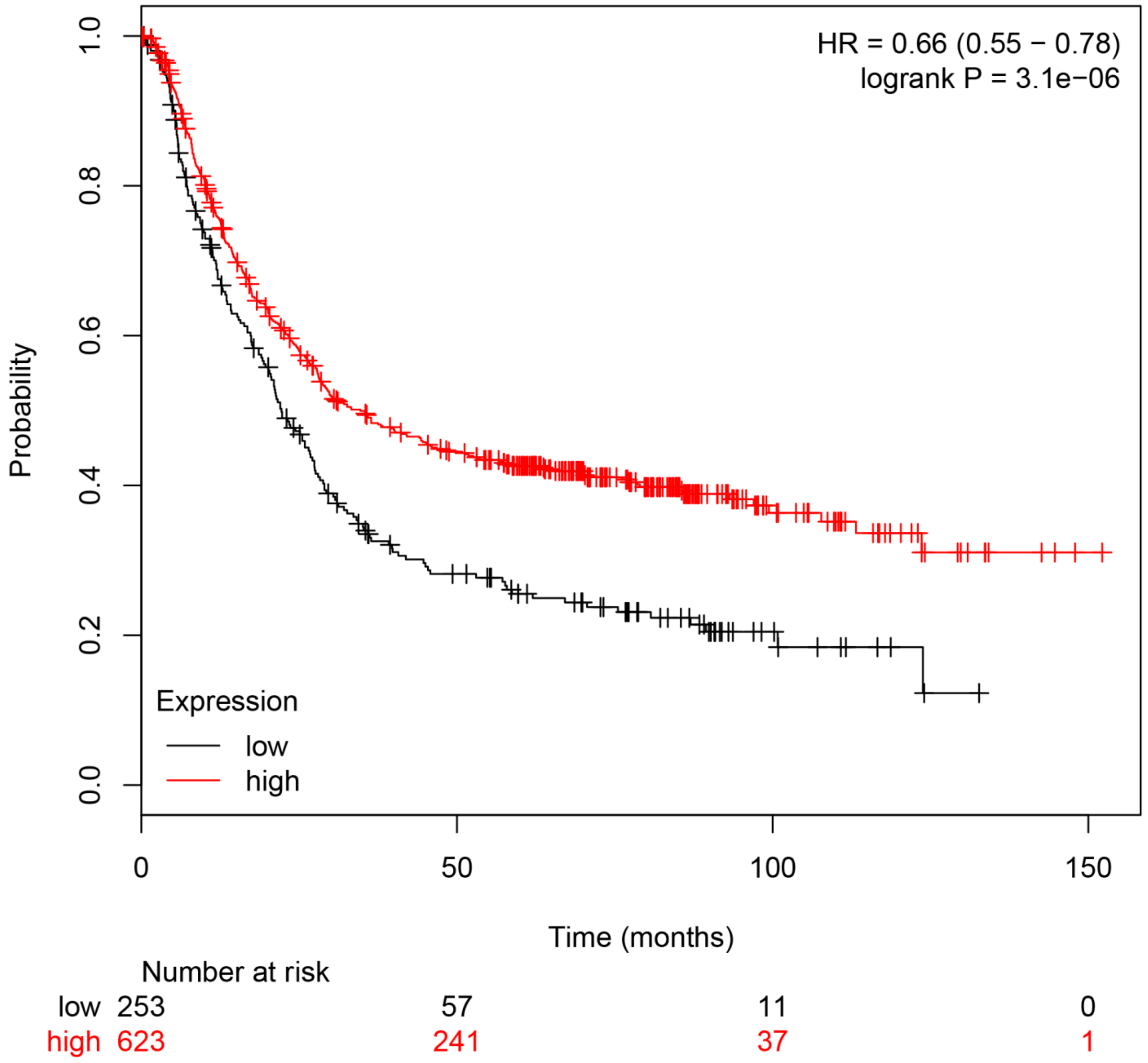

Figure S5: The survival analysis on *MYC*.

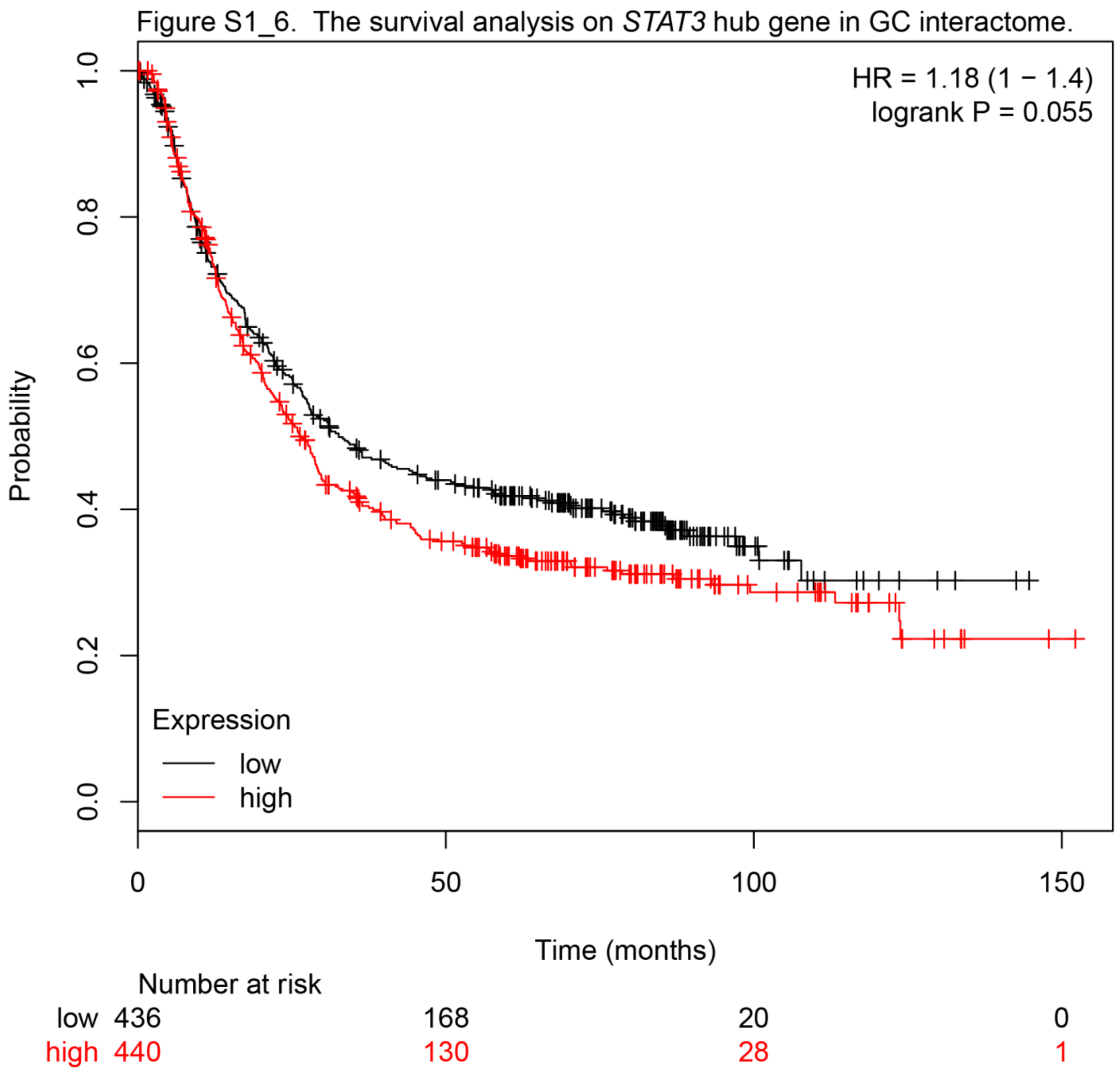

Figure S6: The survival analysis on *ATAT3*.
